# A retrospective on statistical mechanical models for hemoglobin allostery

**DOI:** 10.1063/5.0127585

**Published:** 2022-11-08

**Authors:** William A. Eaton

**Affiliations:** Laboratory of Chemical Physics, National Institute of Diabetes and Digestive and Kidney Diseases, National Institutes of Health, 5/104, Bethesda, Maryland 20892-0520, USA

## Abstract

Understanding allosteric interactions in proteins has become one of the major research areas in protein science. The original aim of the famous theoretical model of Monod, Wyman, and Changeux (MWC) was to explain the regulation of enzymatic activity in biochemical pathways. However, its first successful quantitative application was to explain cooperative oxygen binding by hemoglobin, often called the “hydrogen molecule of biology.” The combination of its original application and the enormous amount of research on hemoglobin has made it the paradigm for studies of allostery, especially for multi-subunit proteins, and for the development of statistical mechanical models to describe how structure determines function. This article is a historical account of the development of statistical mechanical models for hemoglobin to explain both the cooperative binding of oxygen (called homotropic effects by MWC) and how oxygen binding is affected by ligands that bind distant from the heme oxygen binding site (called heterotropic allosteric effects by MWC). This account makes clear the many remaining challenges for describing the relationship of structure to function for hemoglobin in terms of a satisfactory statistical mechanical model.

## I. INTRODUCTION

It is only a moderate exaggeration to draw an analogy to the famous quote of former US President Truman: “There is nothing new in (biochemistry) the world except the history (of hemoglobin) you do not know.” Hemoglobin has been the paradigm for allostery since the seminal paper by Monod *et al.*^1–5^ It is for this reason that I decided to contribute an article to this special issue, because it is not widely known that many of the modern concepts concerning allostery, such as conformational pre-equilibria, were contained in hemoglobin research many decades ago, as pointed out in the excellent review by Qui and Karplus.^6^ Their review of allostery discusses issues for a variety of proteins and is much more comprehensive than what I am about to present. Nevertheless, my retrospective with a somewhat different approach to hemoglobin and allostery should be informative. While many protein crystallographers of my generation concluded that “Max (Perutz) solved the hemoglobin problem,” from my perspective as a biophysical scientist, this could not be further from the truth. Despite well over 100 years of research and almost 200 000 publications concerning hemoglobin, we shall see that very important outstanding problems remain to be solved, especially those closely related to modern-day structural biology and allosteric theory.

In this retrospective, I will focus almost exclusively on the partition function of allosteric models for hemoglobin—the list of the relative populations (Boltzmann weights) of the microscopic states of a model at equilibrium. My reason for doing this is that such a mathematical description is a totally unambiguous description of a theoretical model. The emphasis is, of course, centered on the allosteric model of Monod, Wyman, and Changeux (MWC)^1^ and its elaborations—by far the most influential statistical mechanical model in all of biology. As is documented in the 2013 special issue of the Journal of Molecular Biology, the model has been successfully applied to a myriad of problems in biology.^3^ Because hemoglobin undergoes so many different kinds of chemical reactions and is amenable to study by almost every spectroscopic, biochemical, and physical method, there is an enormous amount of experimental information related to theoretical descriptions of the complex hemoglobin chemistry. Consequently, many of the conclusions made in this article—some of them over-simplified for clarity—will not be justified in detail. I refer the interested reader to several informative reviews on hemoglobin.^7–13^

## II. PAULING’S MODEL

The history of hemoglobin allostery begins with a truly brilliant paper in 1935 by the legendary chemist Pauling.^14^ His objective was to connect a theoretical model of how hemoglobin binds oxygen cooperatively with its structure. In the allosteric language of MWC, to be discussed in detail below, oxygen is a homotropic allosteric effector. Pauling used the tiny bit of information available to him at the time, together with his construction of a statistical mechanical model, to postulate the arrangement of the 4 hemes in the hemoglobin molecule. His 1935 work could therefore be considered the very first example of a structure–function relation in protein chemistry, or what more accurately should be called a function–structure relation. Pauling’s remarkable intellect is evident in his concise description in the introduction of his paper:

“On the basis of certain postulates regarding the structure of the hemoglobin molecule, I have derived an equation involving only two constants, which satisfactorily represents the data on the oxygen equilibrium at constant pH, and also an equation involving two constants, which represents the change in the oxygen equilibrium with change in pH. The validity of these equations provides considerable support for a particular structure of the molecule.”

**FIG. 1.**
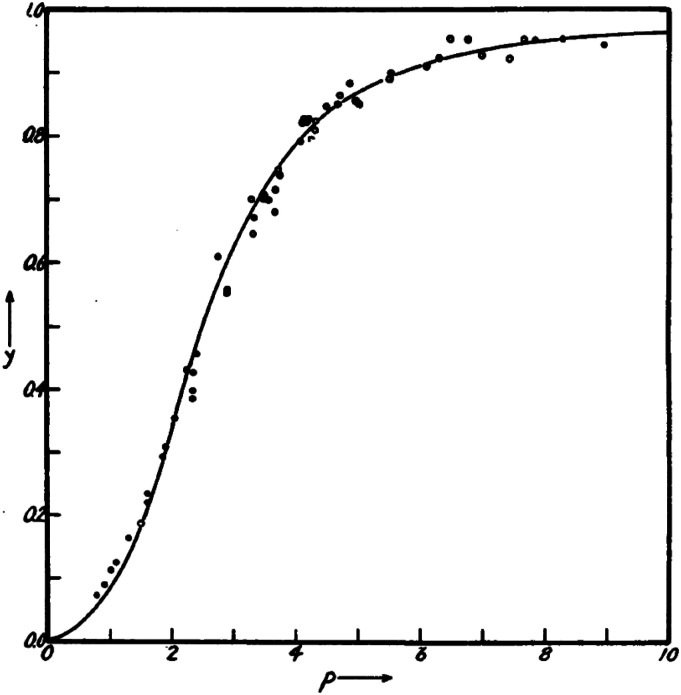
Oxygen binding curve. The fractional saturation with oxygen is *y*; *p* is the oxygen pressure. From Pauling.^14^

**FIG. 2.**
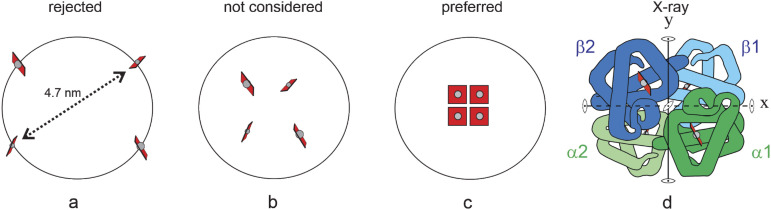
Arrangement of 4 hemes in hemoglobin. (a) A tetrahedral arrangement with the hemes on the surface was rejected by Pauling because the hemes were separated by too large a distance for heme–heme interaction. (b) The structure that Pauling could have proposed if he did not assume that the hemes must be on the molecular surface and did not assume that the hemes must be close together to have a resonance interaction. (c) Pauling’s proposed structure places the hemes close together in a square-symmetric array, presumably to allow for a change in the oxygen-binding heme electronic structure upon oxygen binding from a change in resonance interaction between porphyrin π electrons.^20^ (d) Adaptation of the Dickerson and Geis schematic structure of hemoglobin x-ray structure.^21^ In hemoglobin, αβ dimers are related by an exact twofold rotation axis, **y**. Because of the similarity in the polypeptide fold of the α and β subunits, **x** and **z** have been considered approximate or pseudo twofold rotation axes—the overall symmetry being referred to as pseudo tetrahedral or pseudo 222 symmetry.

Pauling knew that there was about 16 700 g of protein per iron atom from 19th century analytical chemistry and, together with Gilbert Adair’s osmotic pressure measurements, that the molecular weight of hemoglobin is about 66 800 Da.^15^ With 4 hemes per molecule, a protein density of about 0.74 cc/g, and assuming the molecule to be roughly spherical he estimated its diameter to be 5.8 nm. He also reasoned that the 4 hemes are in equivalent environments because he could fit Adair’s oxygen binding curve with a single interaction parameter, *α*, together with an equilibrium constant for oxygen binding, *K*′ [Fig. 1, Eq. (1)],^15^

$$\begin{array}{ll} y\left(p\right) & =\frac{K^{\prime }p\;+\;\left(2\alpha +1\right)K^{\prime 2}p^{2}\;+\;3\alpha^{2}K^{\prime 3}p^{3}\;+\;\alpha^{4}K^{\prime 4}p^{4}}{1\;+\;4K^{\prime }p\;+\;\left(4\alpha +2\right)K^{\prime 2}p^{2}\;+\;4\alpha^{2}K^{\prime 3}p^{3}\;+\;\alpha^{4}K^{\prime 4}p^{4}}\\ & =\frac{1}{4}\frac{d\ln\Xi \left(P\right)}{d\ln p}, \end{array}$$ (1)

where Ξ(P) is the Pauling (P) partition function [Eq. (2)]; this partition function is the macroscopic analog of the grand canonical partition function of Hill and the binding polynomial of Wyman,^16–18^ [Eq. (2)]. In Pauling’s scheme below, Eq. (1) was derived assuming that the hemes are arranged in a square array where the interaction between hemes that increases the affinity for oxygen occurs between adjacent hemes (heavy bars) with no interaction across the diagonal,
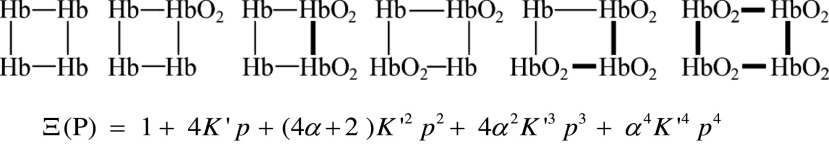
(2)Pauling considered a tetrahedral arrangement of the 4 hemes but rejected it because he assumed that the hemes must be on the molecular surface to bind oxygen. Placing the hemes on the molecular surface in a tetrahedral arrangement would make them too far away (he estimated 4.7 nm) for any significant heme–heme interaction that he was considering as the source of cooperativity. He apparently did not bother to consult any of his organic chemistry colleagues at Cal Tech, who could have immediately told him that hemes exposed to water would not bind oxygen, but instead oxygen would rapidly oxidize the iron of the hemes from Fe(II) to the non-oxygen binding Fe(III). My speculation is that he would not consider placing them inside the protein because, as a crystallographer in the 1930s, he did not envisage that the structures could be sufficiently flexible to allow oxygen to penetrate the protein. In addition, a tetrahedral arrangement of the hemes, even if they were close to each other, may not result in resonance interaction, which was presumably the nature of his heme–heme interaction, as suggested by the last sentence in his paper:“…. in hemoglobin the four hemes are conjugated.” It is not at all obvious why Pauling did not even mention another symmetry arrangement with 4 equivalent hemes = 222 (D_2_), with 3 orthogonal twofold rotation axes, which could have a change in resonance interaction between porphyrin π electrons upon oxygenation. So, Pauling came close to arriving at the correct arrangement of the hemes in the globin [Fig. 2(d)] more than 25 years before Max Perutz determined it from x-ray crystallography.^19^

Pauling then went on to address the origin of the Bohr effect, which is the decrease in hemoglobin oxygen affinity in the tissues due to the lowering of pH from the release of carbon dioxide into the blood.^22^ (Christian Bohr, the physiologist, is the father of physicist Niels Bohr of the Bohr hydrogen atom). Protons and 2,3-diphosphoglycerate (2,3-DPG), the latter unknown to Pauling and discovered after MWC published their paper, are the two major allosteric effectors of hemoglobin that do not bind to the heme (carbon dioxide is another but is not discussed here). In MWC terminology, they are called heterotropic allosteric effectors. As pointed out below, understanding the structural basis of the Bohr effect was a centerpiece of Max Perutz’s famous 1970 paper on the stereochemical mechanism.^23^ Pauling could quantitatively explain the effect of pH on the oxygen affinity with a simple model assuming that each heme interacts with two ionizable residues of the protein to produce a modification of his intrinsic binding constant *K*′, i.e.,

$$K^{\prime }=K\frac{{\left(1+\beta A/\left[H^{+}\right]\right)}^{2}}{{\left(1+A/\left[H^{+}\right]\right)}^{2}},$$ (3)

where *K*, *β*, and *A* are adjustable fitting parameters (−log *A* is the pK = 7.9 of the ionizable groups).

An elaboration of Pauling’s model was proposed in 1966 by Koshland, Nemethy, and Filmer (KNF).^24^ The model utilizes the earlier “induced fit” theory of Koshland that binding induces a conformational change, which is a key element of the Perutz stereochemical mechanism and, more importantly, of modern research on structural mechanisms responsible for allostery.^25,26^ KNF is also a sequential model, with the difference from Pauling’s model being that the change in oxygen affinity results from an interaction between subunits that alters the tertiary conformation of its neighboring subunit when oxygen binds. Besides being an algebraic morass, it is not useful to go into it in any more detail here, since it has been shown from definitive experiments that it only applies to hemoglobin under a very special set of conditions [Eq. (12) below]. I should point out that there is a history of confusion in the hemoglobin field because of the lack of recognition that KNF and induced fit are different—the former being a theoretical model, the latter being a structural concept.

## III. MODEL OF MONOD, WYMAN, AND CHANGEUX

An equally simplistic but radically different model was proposed in the landmark paper by Monod *et al.* (MWC) in 1965,^1^ one of the most highly cited works in all of theoretical biology (7691 times as of October 2022 according to the Web of Science; as a comparison, Watson and Crick’s 1953 *Nature* paper on the structure of DNA has been cited 8360 times). While the statistical mechanical model is presented in the 1965 paper, the key idea is contained in 1963 paper [“The biological activity of many proteins is controlled by specific metabolites, which do not interact directly with the substrates or products of the reactions. The effect of these regulatory agents appears to result exclusively from a conformational alteration (allosteric transition) induced in the protein when it binds the agent.”]^27^ Each of the authors is an extremely interesting individual and a tremendously successful scientist. Monod is generally given credit for being the intellectual force that produced the model, although Wyman’s earlier work provided an important foundation.^28,29^ Monod (1910–1973) was one of the founders of the field of molecular biology and also won the 1965 Nobel Prize for genetic control of enzyme and virus synthesis. He was a leader in the French resistance during World War II, becoming chief of staff of the French Forces of the Interior. Wyman (1901–1995) resigned as Professor of Biology at Harvard at age 50 to become the first scientific attaché at the American Embassy in Paris and moved in 1955 to Cairo to assume the position of regional director of the United Nations Educational, Scientific, and Cultural Organization (UNESCO). In 1958, he visited Antonini in the Department of Biochemistry at the Sapienza University of Rome and liked it so much that he stayed as a visiting scientist until his retirement in 1984, providing a rigorous thermodynamic analysis of the experiments by Antonini, Brunori, and co-workers. Changeux (1936 - ), a Ph.D. student of Monod and Monod’s co-winner of the 1965 Nobel Prize, Francois Jacob, became a famous neuroscientist as a result of his identification and purification of the nicotinic acetylcholine receptor, to which he successfully applied MWC, and for his theoretical work on cognition.^30^ For his neuroscience work, Changeux has received numerous awards and honorary degrees from universities. Changeux is also an art expert, holding senior positions in many different major art organizations.

Wyman and MWC introduced the notion that cooperative effects in proteins arise from conformational changes, altering how we have thought about how proteins function since the 1960s. Monod was interested in the control of metabolism by regulating enzymatic activity and was influenced by three important factors in the development of MWC. One was the experiments of his graduate student, Changeux, which showed that an enzyme could be inhibited by a small molecule bound to other than the active site.^31^ A second was Perutz’s finding that the arrangement of the 4 subunits of hemoglobin is different in oxyhemoglobin and deoxyhemoglobin; i.e., they have different quaternary conformations.^19^ Third, he learned about and was influenced by linked thermodynamic relations and connections to protein conformation that form the foundation of MWC by interacting with Wyman,^29^ who in turn was influenced by the experimental work of Antonini and co-workers.^32^

**FIG. 3.**
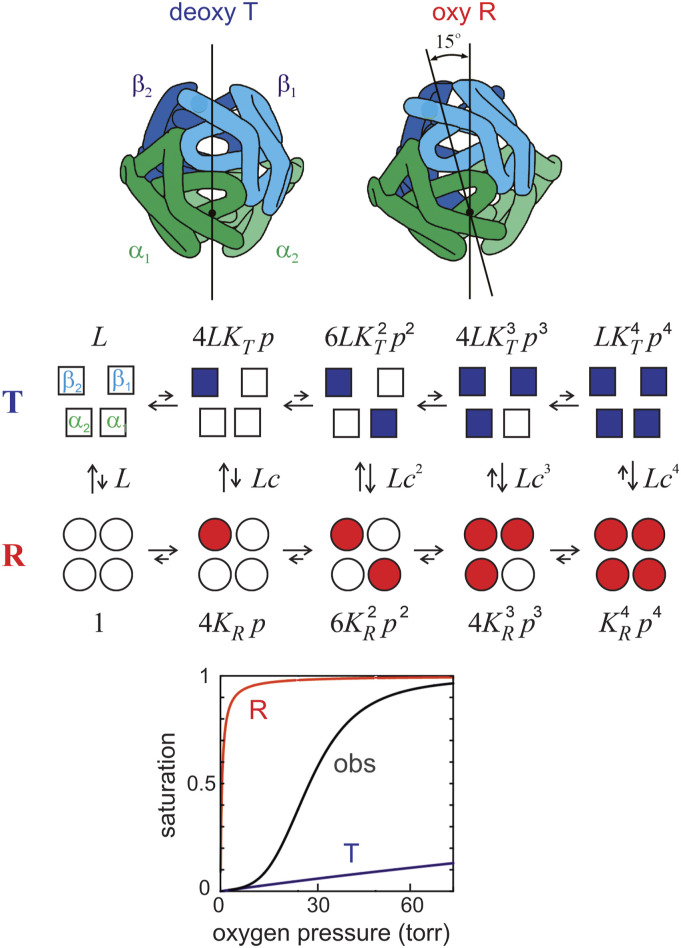
The MWC two-state allosteric model. (Upper panel, quaternary structures) The main difference between the two quaternary structures is a rotation of the symmetrically related αβ dimers of about 15°. In this transition, the interface between α_1_ and β_2_ change, while the interface between α_1_ and β_1_ remains unaltered.^23^ (Middle panel, model) A diagramatic representation of the MWC model. Empty symbols designate deoxygenated subunits and filled symbols oxygenated subunits. *K*_T_ (∼0.001 Torr^−1^) and *K*_R_ (∼1 Torr^−1^) are the association constants for binding to the **T** and **R** quaternary structures, respectively, *L* (∼5 × 10^5^) is the ratio of the **T** to R population at zero oxygen pressure, and *c* = *K*_T_/*K*_R_. The approximate magnitude of the **T-R** equilibrium constant is indicated schematically by the relative length of the arrows for the forward and backward reaction rates. The coefficients for the Boltzmann weights of each ligation species are the statistical factors. (Lower panel, binding curves) The oxygen binding curve for both the **R** and **T** conformations is non-cooperative. The shift from the **T** to **R** conformation as successive molecules of oxygen bind results in the observed (obs) sigmoid shape, signifying cooperativity.

The most important feature of the model for today’s research on allostery is that it postulates a pre-equilibrium between two (or more) conformations of the protein, each one with a different biochemical reactivity/function. The relative population of the conformations is controlled by both homotropic and heterotropic allosteric effectors. In the case of hemoglobin, there are two quaternary conformations; the homotropic effector is oxygen, and the heterotropic effectors are protons and 2,3-DPG. The conformation of the fully deoxygenated molecule was called **T** for tense because of the constraining bonds between subunits, so-called “quaternary constraints,” while the conformation of the fully oxygenated molecule was called **R** for relaxed, implying weaker inter-subunit bonds and predicting that oxyhemoglobin dissociates into αβ dimers much more readily than deoxyhemoglobin, as observed.^8^ Even though hemoglobin has a single axis of symmetry (C2) and only pseudo-tetrahedral (or pseudo D_2_) symmetry, it is used over and over again by MWC, assuming 4 equivalent subunits as an example of many of the points made in the paper. Because it led to years of controversy, a critically important postulate of the model is that binding to each of the quaternary conformations is non-cooperative (confirmed by experiments; see below); cooperative binding, manifested as a sigmoid shape to the oxygen binding curve, results from initial binding to the low affinity **T** conformation and a displacement of the **T**-**R** equilibrium toward the high affinity **R** conformation as successive molecules of oxygen bind (Fig. 3). The key feature of the MWC model relevant to today’s allostery research is the postulate that heterotropic allosteric effectors do not alter the association constants of either **T** (*K*_*T*_) or **R** (*K*_*R*_), but only change the **T-R** equilibrium constant, *L* (=[T_0_]/[R_0_]). The elegantly simple MWC partition function for 4 equivalent subunits [which is the denominator of the MWC state function Eq. (1) in their paper]^33^ is

$$\Xi {\left(MWC\right)}_{\alpha =\beta }=L{\left(1\;+\;K_{T}p\right)}^{4}+{\left(1+K_{R}p\right)}^{4},$$ (4)

where *p* is the oxygen pressure. The saturation function, *y*(*p*), is given by

$$\;y\left(p\right)=\frac{1}{4}\frac{d\ln\Xi (MWC)}{d\ln p}=\frac{LK_{T}p{\left(1+K_{T}p\right)}^{3}+\;K_{R}p{\left(1+K_{R}p\right)}^{3}\;}{L{\left(1+K_{T}p\right)}^{4}+\;{\left(1+K_{R}p\right)}^{4}\;}.$$ (5)

Treating the α and β subunits as inequivalent leads to a simple extension of the MWC partition function to^8,34^

$$\begin{array}{ll} \Xi {\left(MWC\right)}_{\alpha \neq \beta } & =L{\left(1\;+\;K_{T}^{\alpha }p\right)}^{2}{\left(1\;+\;K_{T}^{\beta }p\right)}^{2}\\ & \;+{\left(1\;+\;K_{R}^{\alpha }p\right)}^{2}\;{\left(1\;+\;K_{R}^{\beta }p\right)}^{2}, \end{array}$$ (6)

which does not alter the postulate that binding in each quaternary structure is non-cooperative and that the shift from **T** to **R** as successive molecules of oxygen bind is responsible for cooperativity.

Hemoglobin has only a single true twofold symmetry axis, which interchanges αβ dimers [Fig. 2(d)], so the functional unit of the hemoglobin tetramer is not the individual subunit as assumed in the MWC partition function [Eq. (5)], but is the αβ dimer, which is the “protomer” of MWC. Brunori and co-workers introduced what they called the “cooperon model,”^35^ which should be regarded as the exact MWC model since it recognizes that the molecule has only a single axis of symmetry. The model was introduced to explain the conclusion by Ackers and co-workers that the **T** conformation binds oxygen cooperatively, later shown to be incorrect (see discussion below in the section on Controversy). The exact MWC (cooperon) partition function is

$$\begin{array}{ll} \Xi \left(exact\;MWC\right) & =L{\left[1\;+\;\left(K_{T}^{\alpha }+K_{T}^{\beta }\right)p\;+\;\delta_{T}K_{T}^{\alpha }K_{T}^{\beta }p^{2}\;\right]}^{2}\\ & \;+\;{\left[1\;+\;\left(K_{R}^{\alpha }+K_{R}^{\beta }\right)p\;+\;\delta_{R}K_{R}^{\alpha }K_{R}^{\beta }p^{2}\;\right]}^{2}, \end{array}$$ (7)

where *δ*_*T*_ and *δ*_*R*_ are the increases in affinity upon binding the second ligand to an αβ dimer in **T** and **R**, respectively.

The (approximate) MWC partition function that accounts for binding of heterotropic allosteric effectors, with the simplification that *K*_*T*_ = 0, is

$$\Xi {\left(MWC\right)}_{\alpha =\beta ,het}=L_{0}\frac{{\left(1+K_{I}I]\right)}^{4}}{{\left(1+K_{A}A]\right)}^{4}}+\;{\left(1+K_{R}p\right)}^{4},$$ (8)

where *L*_0_ is the **T**/**R** equilibrium constant in the absence of any heterotropic allosteric effectors, *K*_*I*_ and *K*_*A*_ are the association constants for the inhibitor that binds to **T** and the activator that binds to **R** at concentrations *I* and *A*, respectively. In the case of hemoglobin, there are only inhibitors (protons and 2,3-DPG) and no physiological activators (*Κ*_*A*_ = 0). A modification of Eq. (8) for a simple ligand binding reaction with association constants $K_{C}^{t}$ and $K_{C}^{r}$ for low and high affinity conformation, *t* and *r* respectively, at concentration C is especially important for modern day studies of allostery, which are mostly concerned with single subunit proteins with the two conformations, *t* and *r*, i.e.,

$$\begin{array}{ll} \Xi \left(MWC,monomer\right) & =L_{0}\left(1+K_{I}I\right)\left(1+K_{C}^{t}C\right)\\ & \;+\left(1+K_{A}A\right)\left(1+K_{C}^{r}C\right), \end{array}$$ (9)

with *L*_0_ = *t*/*r* [See Eq. (28) in Ref. 36].

A major postulate of the MWC model is that the symmetry of the multi-subunit molecule (called an “oligomer”) is conserved in the transition between quaternary structures. Its importance to MWC is apparent from the fact that a large part of the paper is spent discussing symmetry, their main point being that the effect of mutations is amplified in symmetrical structures that have the same contacts in each subunit (“isologous” association), thereby providing an evolutionary advantage by providing larger effects from single mutations. The role of tertiary conformations is minimized, as is evident from:“…. it is reasonable to assume that a functionally significant allosteric transition need not involve more than a very small structural alteration of the protomers.” However, no equations were presented that included tertiary conformational changes, so there was no quantitative assessment of their importance.

## IV. MODEL OF SZABO AND KARPLUS

**FIG. 4.**
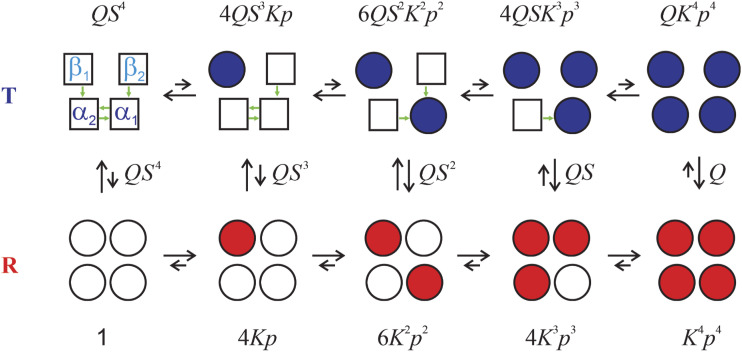
Simplified diagram of the SK model at high pH where all ionizable protons are dissociated to decrease the number of salt bridges in unliganded **T** from 8 to 4. The lower affinity for **T** state subunits results from the energy, *S*, required to break the inter-subunit salt bridges. The allosteric parameter *L* of MWC and *c* correspond to the SK parameters *QS*^4^ and *S*, respectively.

The importance of tertiary conformational changes began with the publication of Perutz’s stereochemical mechanism in 1970, which was the first structural test of the MWC model. Since the initial visualization of the structure showed that hemoglobin is a tetramer of four myoglobin-like subunits, the most interesting finding for Perutz was the inter-subunit salt bridges, which he assigned as ionizable groups that contribute to the Bohr effect. Szabo and Karplus created a statistical mechanical model of Perutz’s mechanism in which there are two quaternary structures, **R** and **T**, and two tertiary structures for the subunits, liganded and unliganded. Figure 4 shows a diagram of the Szabo and Karplus (SK) model.

The only tertiary structural change that is explicitly treated in the SK model is the breakage of salt bridges upon oxygen binding. The SK model is consistent with the MWC postulate that the **T** to **R** quaternary change is required for cooperative oxygen binding. However, it is inconsistent with the MWC postulate that heterotropic allosteric effectors, protons and 2,3-DPG, only affect the quaternary equilibrium constant *L* and not the association constants *K*_*T*_ and *K*_*R*_.^36,37^ Because it addresses structural details and does not treat the α and β subunits as equivalent, their partition function (they called it the “generating function”) is necessarily much more complex,

$$\begin{array}{ll} \Xi \left(SK\right) & =\left\{QS^{6}{\left(1\;+\;\mu H^{\alpha }/S\right)}^{2}{\left(1\;+\;\mu H^{\beta }/S\right)}^{2}\right.\\ & \;\times {\left.{\left[1+\frac{K^{\alpha }\left(1+\mu H^{\alpha }\right)}{S^{2}\left(1+\;\mu H^{\alpha }/S\right)}p\right]}^{2}{\left[1+\frac{K^{\beta }\left(1+\mu H^{\beta }\right)}{S^{2}\left(1+\;\mu H^{\beta }/S\right)}p\right]}^{2}\right\}}_{T}\\ & \;+\left\{{\left(1\;+\;\mu H^{\alpha }\right)}^{2}{\left(1\;+\;\mu H^{\beta }/S\right)}^{2}{\left[1\;+K^{\alpha }p\;\right]}^{2}\right.\\ & \;\times {\left.{\left[1+\frac{K^{\beta }\left(1+\mu H^{\beta }\right)}{S\left(1+\;\mu H^{\beta }/S\right)}p\right]}^{2}\right\}}_{R}, \end{array}$$ (10)

where *S* is the strength of a salt bridge (assumed to be the same strength for the two α1-α2, one α1-β2, one α2-β1 and two intra β salt bridges), *μ* is the OH^−^ concentration, *H*^*α*^ is the hydroxyl binding constant for salt bridges originating from the alpha subunits, *H*^*β*^ is the hydroxyl binding constant for salt bridges originating from the beta subunits, *K*^*α*^ is the intrinsic oxygen association constant for the alpha subunits, *K*^*β*^ is the intrinsic oxygen association constant for the beta subunits, and *p* is the oxygen pressure. For very large values of μ, it is easier to see that the SK partition function simplifies to the same form as the MWC partition function, Ξ(MWC)_α=β_ [Eq. (4)],

$$\begin{array}{c} \Xi {\left(SK\right)}_{\alpha =\beta }=QS^{4}{\left(1\;+\;Kp/S\right)}^{4}\;+\;{\left(1\;+\;Kp\right)}^{4}. \end{array}$$ (11)

The SK partition function is important in several respects. It shows that Perutz’s verbal description of the mechanism is perfectly consistent with cooperative binding arising from the increase in affinity associated with the **T** to **R** transition, as postulated by MWC; from an excellent fit of the pH dependence of the oxygen binding curves, it shows that the salt bridges, the key structural feature of Perutz’s mechanism, can quantitatively account for the Bohr effect; it shows that the inter-subunit salt bridges are plausible candidates for MWC’s quaternary constraints and therefore the origin of the lower oxygen affinity of **T** compared with **R**, as well as the greater stability of the **T** tetramer compared to **R** for dissociation into two αβ dimers. According to SK, breaking salt bridges when ligands bind, in keeping with Koshland’s induced fit theory (not his KNF model), is the key tertiary conformational change. It also makes an important prediction, never discussed in the early work, that deoxy subunits trapped in the liganded conformation of the **T** quaternary structure should have the properties of subunits in the **R** quaternary structure, as observed in the experiments described below in the description of the tertiary two state model.

In a later paper, Szabo and Karplus considered the effect of the heterotropic allosteric effector, 2,3-DPG.^36^ 2,3-DPG binds in a pocket between the two β subunits, which not only shifts the **T-R** equilibrium toward **T**, consistent with MWC, but also alters the affinity of the β subunits in **T** for oxygen, which is a major inconsistency with MWC, presumably by altering tertiary conformation. According to MWC, heterotropic effectors only change *L*, with no effect on *K*_*T*_ or *K*_*R*_. At constant pH, the SK partition function becomes [Eq. (31) in their 1976 paper]^36^

$$\begin{array}{ll} \Xi {\left(SK\right)}_{\alpha \neq \beta ,DPG} & =L_{0}\left(1+\nu P_{\text{T}}\right){\left[1\;+\;K^{\alpha }p/S\right]}^{2}\\ & \;\times \left[1\;+\;2K^{\beta }p/S\left(\frac{1+\nu P_{{\text{T}}^{\prime }}}{1+\nu P_{\text{T}}}\right)\;+\;{\left(K^{\beta }p/S\right)}^{2}\right.\\ & \;\times \left.\left(\frac{1+\nu P_{{\text{T}}^{\prime \prime }}}{1+\nu P_{\text{T}}}\right)\right]\\ & \;+\;\left(1+\nu P_{\text{R}}\right){\left(1\;+\;K^{\alpha }p\right)}^{2}{\left(1\;+\;K^{\beta }p\right)}^{2}, \end{array}$$ (12)

where *ν* is the concentration of 2,3-DPG, *L*_0_ is the **T**/**R** population ratio with no 2,3-DPG bound, *P*_T_, *P*_T′_, and *P*_T″_ are the 2,3-DPG association constants to the **T** quaternary structure when zero, one, and two oxygen molecules are bound to the β subunits, and *P*_R_ is the association constant for 2,3-DPG binding to the **R** quaternary structure, although binding to **R** has not been directly observed. Szabo and Karplus pointed out that this partition function does not have the MWC form, implying a functional interaction between subunits in the **T** conformation as in the KNF model, although the strength of this interaction was not estimated.

Energy calculations by Gelin and Karplus suggested that the low affinity of the **T** conformation is not the result of strain in the deoxy state, as envisaged by MWC and assumed by Perutz to be caused by the salt bridges, but is at least in part due to the strain in the oxygenated **T** confromation from repulsive interactions within the heme–oxygen complex and surrounding residues that they called the “allosteric core.”^38,39^ This finding motivated Lee and Karplus to formulate a generalization of the SK model to include the contribution of the strain to **T** state affinity, as well as the inequivalence of the inter-subunit and intra-subunit salt bridges.^40,41^ The result is only a small difference with the SK partition function [Eq. (10)], consisting of two additional parameters, so it is not presented here.

## V. YEARS OF CONTROVERSY

The validity of the most important elements of the MWC model for explaining cooperative oxygen binding by hemoglobin was a controversial subject for more than 30 years. Since modern day allostery researchers are most probably not interested in the history of hemoglobin controversies, I will not go into any details, but provide just a few summary statements (see Eaton *et al.*^11,13,42^ for more extensive discussion). By 1975, Shulman *et al.* had provided rather convincing evidence from their NMR data as well as from their insightful analysis of the kinetics results of Gibson, Antonini, and Brunori that both equilibrium and kinetic results are consistent with MWC.^9,43,44^ In addition, Edelstein had shown that the changes in the Hill cooperativity parameter, *n*, under different conditions and for hemoglobin mutants could readily be explained by the MWC model but not by a KNF model.^8,45^ However, Gibson, the inventor of the stopped flow method and the master of experimental hemoglobin kinetics, concluded from his 1976 CO rebinding and spectral measurements with pulsed microsecond laser pulses and probing at a wavelength isosbestic for ligand binding, reflecting conformational changes, that his results were inconsistent with MWC.^46^ Gibson’s conclusion was challenged by Hofrichter, Henry, and the author, who used nanosecond pulses with multi-wavelength detection to show, with ∼100-fold better time resolution than Gibson’s, that, once stretched exponential tertiary conformational changes are included, the kinetics are consistent with MWC.^47^

A much bigger monkey wrench was thrown by Ackers, who used tetramer-dimer dissociation measurements and thermodynamic linkage relations to predict oxygen binding curves and populations of intermediate species partially saturated with oxygen. He concluded that there was as much cooperativity of binding within the **T** quaternary conformation as that which resulted from the **T** to **R** conformation change,^48^ in contrast to MWC, where both **T** and **R** bind non-cooperatively. The conclusion of Ackers was not readily accepted by the hemoglobin aficionados and his analysis was immediately challenged.^49^ However, Ackers views were so influential that the biochemical community dismissed the application of the MWC model to hemoglobin for several years (MWC was dropped from many major biochemistry textbooks), apparently because of his seemingly rigorous thermodynamic analysis (in extremely complicated papers, which most scientists could not possibly fully understand without an enormous effort; my colleague Hofrichter described them as “Ackers translates his experimental results into church Latin.”). Experiments and analysis by Edelstein were instrumental in showing that Ackers’ work was simply wrong because it contained artifacts from both electron exchange between hemes and the exchange of hemes between the tetramers.^50^ In addition, Morimoto and co-workers showed that the predicted oxygen binding curves by Ackers from his linkage relations for easily prepared key and stable metal substituted hemoglobins are inconsistent with the directly measured curves,^51^ which Ackers must have measured but never reported. The most convincing experimental evidence refuting the Ackers claim of cooperative binding to **T** came from the use of a microspectrophotometer by Mozzarelli and co-workers to show that oxygen binding to hemoglobin in single crystals, known from x-ray crystallography to remain in the **T** quaternary structure when oxygen bounds is non-cooperative.^52,53^ Their experiments showed that *δ*_*T*_ in Eq. (7) could not be more than 3, indicating that cooperativity within the αβ dimer can be neglected (i.e., *δ*_*T*_ = *δ*_*T*_ = 1 is a reasonable approximation). To complete the story, Shibayama discovered that the quaternary structures could be maintained by encapsulating hemoglobin in silica gels, which showed that both the **R** and **T** conformations bind oxygen non-cooperatively.^54^ Mozzarelli further showed that the Bohr effect in binding to **T** in the Shibayama gels is the same as in free solution,^55^ dismissing the possibility that gel encapsulation introduces artificial constraints.

## VI. TERTIARY TWO-STATE MODEL OF HENRY *et al.*

As pointed out above, the major failure in the MWC model for hemoglobin is that protons and 2,3-DPG not only change the allosteric constant *L*, but also change the affinity of the **T** state, *K*_*T*_ (quoting from MWC: “…. as is well known, the oxygen saturation curves obtained at different values of pH can all be superimposed by a simple, adequately chosen, change of the abscissa scale. In terms of the model, this would mean that the binding of the “Bohr protons” does not alter the equilibrium between the two hypothetical states of the protein. Hence, the Bohr protons also themselves would *not* be allosteric ligands….”). The conclusion of MWC was based on the incorrect assumption that the Hill cooperativity parameter *n* is independent of pH. Moreover, the symmetry postulate of MWC required that tertiary conformational changes are only associated with the change in quaternary structure. Symmetry is broken in both the SK model and its generalization by Lee and Karplus, which postulate that within each quaternary structure there are two tertiary conformations for individual subunits, one with and one without oxygen bound.

**FIG. 5.**
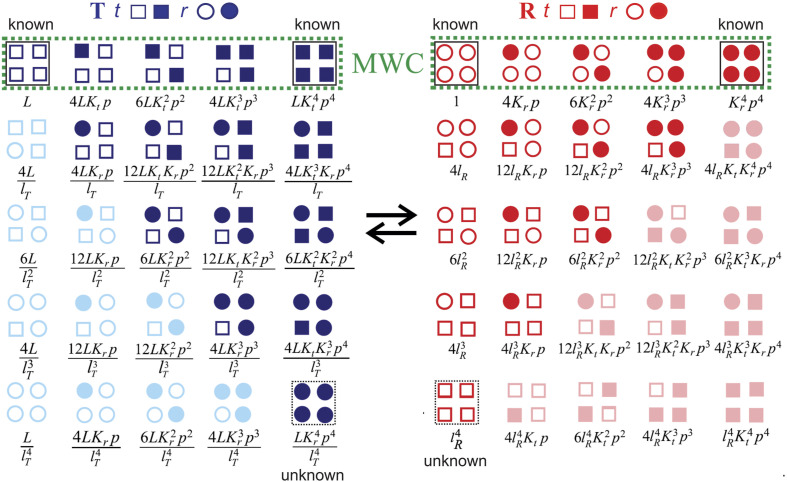
Diagrammatic representation of the TTS allosteric model for equivalent subunits with Boltzmann weights [Eq. (13)].^57^ Approximate values for the parameters of the TTS model are: *L* = 10^6^, *l*_*T*_ = 200, *l*_*R*_ = 0.3, *K*_*t*_ = 0.02, *K*_*r*_ = 5. Subunits with no oxygen bound are represented by open symbols, while oxygenated subunits are represented by filled symbols; squares correspond to the ***t*** tertiary conformation; circles correspond to the ***r*** tertiary conformation; blue tetramers (left) are in the **T** quaternary structure; red tetramers (right) are in the **R** quaternary structure. Each column contains diagrams representing configurations that differ in the number of ligands bound, whereas each row differs in the number of ***t*** and ***r*** tertiary conformations. The first row (enclosed in green boxes) corresponds to the MWC model. Conformations containing oxygenated subunits in the ***t*** conformation in **R** and deoxygenated subunits in the ***r*** conformation in **T** are shown in a lighter color because their populations are predicted to be so small that they can be neglected. In both the MWC and TTS models, ligand binding shifts the quaternary population toward **R**. In the MWC model, the tertiary conformation is completely coupled to the quaternary structure (**T** contains all squares; **R** contains all circles), whereas in the TTS model, the **T** quaternary structure shifts the tertiary population toward ***t*** and the **R** quaternary structure shifts the tertiary population toward ***r***. In both the MWC and TTS models, there are only two ligand-binding equilibrium constants, *K*_*T*_ and *K*_*R*_ in MWC and *K*_*t*_ and *K*_*r*_ in TTS. Enclosed in black boxes with continuous lines are structures that are known from x-ray crystallography. Two key structures to be determined are shown enclosed in boxes with dashed lines.

The tertiary two-state (TTS) model that I am about to describe incorporates the SK notion of two tertiary conformations within each quaternary structure, the difference being that two tertiary conformations can exist with and without oxygen bound, so four tertiary conformations can co-exist within each quaternary structure. The TTS model represents the simplest possible way of extending the MWC model to include tertiary conformational changes upon oxygen binding without a change in quaternary structure. According to the model (Fig. 5), there are two tertiary conformations in each quaternary structure, called ***t*** and ***r***, where ***t*** has the same affinity for oxygen in **T** and **R**, and ***r*** has the same affinity for oxygen in **T** and **R**. The relative population of tertiary structures is biased by both the ligation state and the quaternary structure, with **T** and no oxygen bound favoring ***t***, while **R** and oxygen bound favoring ***r***. In the TTS model, there is a pre-equilibrium of tertiary conformations in addition to the pre-equilibrium of quaternary conformations. The partition function for this model is

$$\begin{array}{ll} \Xi {\left(TTS\right)}_{\alpha =\beta } & =\frac{L}{{\left(l_{T}\right)}^{4}}{\left[1\;+\;K_{r}p\;+\;l_{T}\left(1\;+\;K_{t}p\right)\right]}^{4}\;\\ & \;+{\left[1\;+\;K_{r}p\;+\;l_{R}\left(1\;+\;K_{t}p\right)\right]}^{4}, \end{array}$$ (13)

where *L* is the **T**/**R** population ratio, in which all the subunits of **T** are unliganded ***t*** and all the subunits of **R** are unliganded ***r***, *l*_*T*_ is the ***t***/***r*** tertiary population ratio of unliganded subunits in the **T** quaternary structure, and *l*_*R*_ is the ***t***/***r*** tertiary population ratio of unliganded subunits in the **R** quaternary structure. *K*_*t*_ is the oxygen association constant for ***t***, and *K*_*r*_ is the oxygen association constant for ***r***.^56^

In this model, heterotropic allosteric effectors can alter *L*, *l*_*t*_, and *l*_*r*_, but do not alter *K*_*t*_ or *K*_*r*_*.* Including them explicitly, as in Eq. (12), would lead to a very complex expression. However, Eq. (13) is valid at constant pH without heterotropic effectors or with saturating effector concentrations, as in the experiments of Viappiani and co-workers discussed below, assuming that the effector-binding free energy is linearly proportional to the fraction of ***t*** or ***r*** subunits. The original motivation for the model was (i) the observation in nanosecond-resolved optical spectra, where both **T** and **R** are populated, that there are only two unliganded heme spectra and therefore only two different protein conformations,^58^ (ii) the finding that, in the absence of heterotropic allosteric effectors, the oxygen affinity of **T**-state hemoglobin in single crystals is much lower than the affinity of the **T**-state in solution,^52^ and (iii) the finding that there is no Bohr effect on oxygen binding to **T** crystals.

The key experimental results that could only be explained by the TTS model and none of the models discussed above are the photodissociation experiments at the University of Parma with hemoglobin trapped in either the **T** or **R** quaternary structure by encapsulation in silica gels.^57^ The striking result was that pulsed nanosecond photodissociation of hemoglobin carbon monoxide complex (HbCO) trapped in the **T** quaternary structure showed a fraction of subunits that rebind carbon monoxide at exactly the same rate as found for photodissociation of HbCO in the **R** quaternary structure.^59^ In retrospect, as mentioned earlier, one of the unrecognized predictions of the SK model is that hemoglobin with CO bound in the **T** quaternary structure has broken salt bridges, and therefore 100% of the photodissociated subunits trapped in **T** should bind CO with an **R**-state rate. According to the TTS model, both ***t*** and ***r*** conformations are comparably populated in **T** with CO bound, neatly explaining the result.^59^ In a more demanding experiment by Viappiani and co-workers, a CW laser was used to continuously photodissociate HbCO in the **R** quaternary structure to allow ***r*** to relax to ***t***,^60^ which was evident by the appearance of subunits with **T**-state rebinding rates.^61^ The one caveat in the Parma experiments is that they have all been concerned with CO binding rates, assuming that CO binding rates faithfully reflect CO affinity;^62–64^ future experiments should be focused on measuring CO dissociation rates to obtain equilibrium constants for binding to ***t*** and ***r*** in both **T** and **R**. Such experiments could be performed by measuring the rate of nitric oxide binding to hemoglobin with CO bound encapsulated as either **T** or **R**, since the binding rate will be determined by the CO dissociation rate.^65,66^ Using NO binding to measure the dissociation rates for ***r*** in **R** and for ***t*** and ***r*** in **T** should be doable, while determining the dissociation rate for ***t*** in **R** would require a difficult elaboration on measuring the already difficult experiment of measuring the CO binding rate for ***t*** in **R**.^55^

Interestingly, in both QM/MM and resonance Raman studies of hemoglobin oxygen binding to discover the structural features responsible for high and low oxygen affinity, both high and low affinity tertiary structures are observed to coexist within each quaternary structure, supporting the TTS model.^67,68^

## VII. REMAINING CHALLENGES

The ideal statistical mechanical model is one in which the parameters of the model can be associated with structural features, as in the models of Szabo and Karplus and Lee and Karplus. These models assumed that the two tertiary conformations in each quaternary structure corresponded to the unliganded and liganded states of the subunit. In contrast, the TTS model postulates that there are four tertiary structures for each subunit in both **T** and **R** quaternary structures; unliganded ***t***, unliganded ***r***, liganded ***t***, and liganded ***r***, although the populations of unliganded ***r*** in **T** and liganded ***t*** in **R** are negligible, as indicated by the faded colors in Fig. 5. The structural picture required for interpreting the five parameters of the TTS model (*L*, *l*_*T*_, *l*_*R*_, *K*_*t*_, *K*_*r*_), therefore, will require solving the structures of liganded ***r*** in **T** and unliganded ***t*** in **R**. To construct a partition function including heterotropic allosteric effectors, where all of the parameters can be associated with structural features, will require both additional structures and additional experiments to determine all of the binding constants. There also remains the problem, of particular interest to modern day allostery research, of determining the structural pathways responsible for connecting the heterotropic binding sites and the heme—the active site of hemoglobin. The complexity becomes even greater as the α and β subunits must be considered separately. It should be clear from this brief discussion of remaining challenges that, while Max Perutz made an enormous contribution to hemoglobin and structural biology in general, he “did not solve the hemoglobin problem.”

## Data Availability

The data that support the findings of this study are available within the article.

## APPENDIX: A FEW PERSONAL RECOLLECTIONS OF PERUTZ

Hemoglobin was the most important molecule in protein science during the 1970s, beginning with Perutz’s 1970 paper on his stereochemical mechanism in *Nature*. His paper, which introduced structure–function thinking at the atomic level, attracted a large number of extremely smart scientists to add to the luminaries already working on hemoglobin—Antonini, Brunori, Edsall, Gibson, Monod, and Wyman. The newcomers included Bunn, Edelstein, Hopfield, Karplus, McConnell, Shulman, and Szabo. I am fortunate to have known all of the above, except Monod. Since Perutz is such a central figure in my story and quite a remarkable scientist, I thought the reader may find a few personal anecdotes interesting.

I first met Perutz in the summer of 1962 when I was a medical student working on protein biosynthesis in the group of Brenner. Listening to Brenner and his pal Crick define the important problems in biology in discussions at morning coffee, lunch, and afternoon tea in the canteen of the new MRC Laboratory of Molecular Biology (LMB) in Cambridge, run by Perutz’s wife Gisela, convinced me that I should pursue a Ph.D. after medical school and a life of basic research. That summer was the first of many occasions where I witnessed the human side of Perutz. As director of LMB, he made a point of periodically discussing the research for at least 30 min with everyone working in the lab, including the most minor temporary members, such as me. There was a lot of excitement in the air that summer, with the anticipation of possible Nobel Prizes—Perutz, Kendrew, and Crick all won Nobel Prizes in the fall of 1962. The LMB at that time was a singularity in the history of science, with 5 of the 6 group leaders in 1962—Perutz, Kendrew, Brenner, Crick, and Sanger—winning six Nobel Prizes. Fred Sanger won two and Hugh Huxley none. I also met Shulman that summer, when we played for the LMB cricket team in the annual match against the Department of Biochemistry. We have been good friends ever since.

My meeting with Perutz in 1962 was the first of many for the next several decades. The most important one is when I sent him a letter in December 1972, telling him that I had measured polarized optical absorption spectra of single sickled cells to obtain the orientation of the hemes in the sickle fibers using a microspectrophotometer that I had constructed at NIH. I pointed out to him that I had seen a preprint of his PNAS article on the structure of the sickle fiber from electron microscope measurements with Finch and that the structure could not possibly be correct because the twofold rotation axis normal to the fiber axis placed the β6 valine in a position facing the solvent with no possibility of intermolecular contact within the fiber. His reply was a letter with an all-expenses-paid invitation to present my results at his hemoglobin meeting at the Royal Society in London in February 1973. How many scientists organizing a meeting today would invite a totally unknown scientist to their major international meeting to present work that pointed out what amounted to their blunder?

Perutz’s wife, Gisela, had a brother who lived near Bethesda, which provided many occasions for me to host him for seminars and discussions at NIH. In 1980, I organized a dinner for a group of scientists in the large dining room at the elegant Stone House mansion on the NIH campus. Rather than sit next to one of a number of well-known senior NIH scientists at the dinner, Perutz insisted on sitting next to my 11 year-old daughter Helen and spent the entire dinner asking her about her violin and her studies at The Sidwell Friends School. His behavior that evening was consistent with his reputation as a parent. Unlike many scientists, Perutz was known for putting family ahead of his work and would immediately stop whatever he was doing in the lab if he were needed at home by one of his children.

Perutz was very appreciative of that evening because Frank Bunn, who was a Fogarty-Scholar-in-Residence at the time, entertained with an after-dinner performance of what Frank knew was Perutz’s very favorite piece of music—Mozart’s Kegelstatt Trio, with Frank on the piano, Helen on the violin, and the wife of Helen’s violin teacher on the viola.

Perutz was a tough-minded and fiercely competitive scientist. This side of his character was evident from his critical remarks at many meetings about anything that even slightly contradicted or questioned any aspect of his stereochemical mechanism, especially his proposal that the salt bridges are responsible for the Bohr effect. The reason for his sensitivity about the salt bridges is allegedly the story that his initial impression of the hemoglobin structure was a disappointment because it appeared to be simply 4 myoglobin-molecules assembled into a tetramer. So, identifying the ionizable salt bridges between and within the subunits as the structural origin of the Bohr effect was regarded by him as possibly the most important discovery derived from the structure. On a visit to the NIH in the late 1990s, Brenner told me that Perutz spoke so often about the relationship of the salt bridges in his x-ray structure to the Bohr effect that “Francis and I called it Max’s boring effect.”

I found one example of his attachment to his beloved salt bridges quite amusing. It was when I visited him at the MRC in the 1980s, during a period when Ho was publishing papers using NMR to challenge his assignments of the Bohr amino acid residues.^12,69^ When I entered his office, the first words out of his mouth were not “hello Bill,” but “Bill, I am ***hopping mad*** at Chien Ho.”
